# An Improved Multi-Scale Feature Extraction Network for Rice Disease and Pest Recognition

**DOI:** 10.3390/insects15110827

**Published:** 2024-10-23

**Authors:** Pengtao Lv, Heliang Xu, Yana Zhang, Qinghui Zhang, Quan Pan, Yao Qin, Youyang Chen, Dengke Cao, Jingping Wang, Mengya Zhang, Cong Chen

**Affiliations:** 1 Key Laboratory of Grain Information Processing and Control, Henan University of Technology, Ministry of Education, Zhengzhou 450001, China; pengtaolv@163.com (P.L.);; 2 Henan Key Laboratory of Grain Storage Information Intelligent Perception and Decision Making, Henan University of Technology, Zhengzhou 450001, China; 3 Henan Grain Big Data Analysis and Application Engineering Research Center, Henan University of Technology, Zhengzhou 450001, China; 4 College of Information Science and Engineering, Henan University of Technology, Zhengzhou 450001, China; 5 School of Automation, Northwestern Polytechnical University, Xi’an 710072, China

**Keywords:** rice diseases and pests, deep learning, multi-scale feature extraction, data augmentation, pest image classification

## Abstract

**Simple Summary:**

Rice is one of the most important sources of food for humans. However, rice production is frequently threatened by pests and diseases, resulting in significant losses. In this study, we developed a model that can assist agricultural practitioners in accurately identifying different types of rice pests and diseases. Our model can accurately identify seven different categories of rice pests and diseases, which enables agriculturalists to promptly identify the causes of crop damage and take appropriate measures to protect their crops. We hope that the application of this technology will reduce global rice losses and alleviate the problem of the global food crisis.

**Abstract:**

In the process of rice production, rice pests are one of the main factors that cause rice yield reduction. To implement prevention and control measures, it is necessary to accurately identify the types of rice pests and diseases. However, the application of image recognition technologies focused on the agricultural field, especially in the field of rice disease and pest identification, is relatively limited. Existing research on rice diseases and pests has problems such as single data types, low data volume, and low recognition accuracy. Therefore, we constructed the rice pest and disease dataset (RPDD), which was expanded through data enhancement methods. Then, based on the ResNet structure and the convolutional attention mechanism module, we proposed a **Lightweight Multi-scale Feature Extraction Network (LMN)** to extract multi-scale features at a finer granularity. The proposed LMN model achieved an average classification accuracy of 95.38% and an F1-Score of 94.5% on the RPDD. The parameter size of the model is 1.4 M, and the FLOPs is 1.65 G. The results suggest that the LMN model performs rice disease and pest classification tasks more effectively than the baseline ResNet model by significantly reducing the model size and improving accuracy.

## 1. Introduction

Rice has been a crucial food crop for humans for thousands of years. More than half of the world’s population relies on rice as their primary source of sustenance [1]. Enhancing rice yield and quality is a significant objective of global food production. However, rice is plagued by various pests and diseases during cultivation. The Food and Agriculture Organization of the United Nations [2] reports that plant pests and diseases result in a 20% to 40% loss in global crop production annually. Moreover, global warming and rapid urban expansion have led to the shrinkage of global arable land [2,3]. The global food crisis is becoming more and more serious, and we must take appropriate measures promptly. The global food crisis can be mitigated by effectively identifying and preventing rice pests and diseases, thereby improving rice yield and quality.

The traditional identification of pests and diseases relies mainly on experienced farmers or experts consulting pest maps [4]. Farmers can directly identify and diagnose ordinary pests and diseases. However, the identification of uncommon or specialized pests and diseases requires a high degree of professionalism and rich experience. Manual methods not only take a lot of people’s time and effort but also easily lead to misjudgment. In order to solve such problems, research on accurate and automatic identification methods for crop pests and diseases is the development goal of smart agriculture. Excellent identification technologies can ensure the timeliness of pest and disease detection and reduce the crop yield gap between different countries due to technical barriers.

In recent years, there has been continuous development of deep learning models in the field of image recognition. Convolutional neural networks, such as LeNet [5], VGG [6], GoogLeNet [7], and ResNet [8], have achieved good performance in image classification tasks and have improved the understanding and application of neural networks. More and more scholars are investing time into research on rice pest identification networks. Research methods and model structures are constantly evolving. Huang et al. [9] created a dataset for detecting rice ear blast, using the GoogLeNet model to achieve an accuracy rate of 92%. Xiao et al. [10] classified six types of rice pests under laboratory conditions using a convolutional neural network and transfer learning. Fan et al. [11] collected over 20,000 pictures of rice diseases and pests to build a complete dataset. They developed a deep-learning model for 16 major rice diseases and insect pests using a pre-trained ResNet50 model. In addition, they constructed a dataset for training the filtering model to avoid the misclassification of non-rice pest images.

The traditional convolutional neural network-based rice disease and pest identification model can extract characteristics of diseases or insect pests well, but there are still some shortcomings in the existing research. First, the categories of diseases and pests used by researchers are relatively limited, and the backgrounds of the pictures are simple. Studies on the multi-category classification of rice diseases and pests in complex field backgrounds are still relatively lacking. Secondly, the characteristic parameters of rice diseases and pests are complex, and the existing models have limited generalization ability. The existing models may not accurately extract the characteristics of diseases and pests in the pictures. They tend to extract many irrelevant features from the pictures, which easily leads to overfitting and poor effectiveness of the models. Finally, the practical application of rice pest and disease recognition models is relatively limited. Most of the research only remains in the theoretical stage without practical application. In other words, the existing models are too large and not suitable for deployment on mobile devices and websites.

To tackle the aforementioned concerns, we proposed a new and innovative model called the Lightweight Multi-Scale Feature Extraction Network (LMN). We also deployed the proposed model to a website to provide pest and disease identification services. Based on the ResNet18 structure, our model enhances the receptive fields of each network layer. It can extract multi-dimensional features of disease and pest characteristics from a fine granularity perspective. It is helpful in accurately identifying types of rice diseases and pests with complex backgrounds. Moreover, our proposed lightweight convolutions reduce the complexity of the model, making it suitable for application. In summary, this paper presents the following primary contributions:

(1) We constructed a rice pest and disease dataset (RPDD). The RPDD has more complex backgrounds and more abundant categories. We collected 21,072 rice pest and disease images, and the images were divided into seven categories. The dataset was augmented and expanded by data enhancement methods, including geometric transformation, color transformation, and the CutMix augmentation strategy.
(2) We proposed a new lightweight model (LMN) for rice pest and disease recognition. The LMN can extract multi-scale features of disease and pest characteristics at a fine granularity level to improve accuracy. The LMN incorporates a new lightweight module (i.e., the SM module) based on the improved ResNet18, and the complexity is reduced. Compared to conventional convolutional neural networks, our proposed model can effectively and efficiently identify rice pests and diseases with complex backgrounds and prevent overfitting with low parameters.
(3) We conducted extensive experiments on the constructed dataset to assess the performance of the proposed model. In the experiments, our LMN model achieved an average recognition accuracy of 95.38%, and the parameter size was 1.4 M. This demonstrates the excellent performance of our model.

In addition, we have created a mobile applet application to ensure the implementation of our model. This deployment allows users to conveniently utilize our model in their studies and investigations related to identifying rice pests and diseases.

The rest of the paper is organized as follows. Section 2 describes related studies and introduces some feature extraction methods. Section 3 describes our proposed rice disease and pest classification model, including the improvement of the base model and the proposed lightweight SM module. Section 4 describes the dataset, data enhancement methods, experimental setup, evaluation metrics, and experimental results. Section 5 concludes the paper and presents our future work.

## 2. Related Studies

### 2.1. Pest and Disease Classification Methods and Models

Traditional machine learning methods mainly rely on human intervention for feature extraction and classifier selection to achieve classification results related to rice pests and diseases. For example, Wang et al. [12] used backpropagation (BP) networks as classifiers for grape and wheat diseases, respectively. Padol et al. [13] used SVM classification techniques for the detection of grape leaf diseases. However, due to the diversity of structures and the limited generalizability of specific models, much research may not be applied to practical scenarios. As artificial intelligence (AI) continues to develop, deep learning has been widely applied to complex agricultural scenarios. Amara et al. [14] used CNN-based LeNet and image processing to identify two types of leaf diseases. Rahman et al. [15] proposed a two-stage training-based CNN rice pest and disease detection model to classify images of rice pests and diseases. Mohanty et al. [16] employed a deep CNN model to classify 14 crops with 26 diseases. A novel CNN-based rice disease detection method for detecting various diseases in rice was proposed by Yang et al. [17]. Chen et al. [18] classified rice diseases using transfer learning and VGGNet. They also developed a new model called SE-MobileNet to identify different plant diseases [19]. However, the above studies mainly focused on the accurate identification and classification of plant diseases. They overlook reducing the complexity of models. Therefore, we aim to minimize the size of the model parameters while achieving a balance between complexity and recognition accuracy.

### 2.2. Attention Mechanism for Lightweight Network

In the field of convolutional neural networks, the channel attention mechanism has been an important area of research. The network can extract key features by dynamically learning the information of each channel. Many researchers have proposed effective lightweight channel attention mechanisms. In 2018, Woo et al. [20] proposed CBAM, which combines channel attention and spatial attention. CBAM compresses the feature maps in both the channel and spatial dimensions. A two-dimensional feature map is obtained by merging different feature maps. CBAM can adapt well to feature optimization by multiplying the input feature maps. Hu et al. [21] proposed the SE attention mechanism in 2017, which gives different channels of the image varying weights by introducing the squeeze operation and the excitation operation. Hence, the model can focus on the more important channel features. Wang et al. [22] improved the SE attention mechanism in 2019. Using the one-dimensional convolution technique, the authors achieved a local cross-channel interaction methodology without reducing dimensionality. Hou et al. [23] proposed the coordinate attention mechanism as an effective attention mechanism for lightweight networks. The network can access information from a larger scope while avoiding high costs.

### 2.3. Receptive Field Enhancement

Our goal in designing the rice pest and disease identification model is to take advantage of different structures to reduce the parameter size of the model while ensuring classification accuracy. In this section, we present two methods of sensory field enhancement in rice pest and disease identification tasks: (1) Dilated convolution: to avoid information loss caused by the reduction of image resolution during the down-sampling process, Wei et al. [24] proposed dilated convolution for image segmentation. Dilated convolution, which introduces a hyperparameter known as the dilation rate, is added to the traditional convolutional layer. Dilated convolution allows a larger receptive field and more information without introducing additional computational cost. Meanwhile, it can preserve the internal data structure of the original image. The other method is (2) inception architecture, which is a method of aggregating different receptive fields proposed by Szegedy et al. [7]. Inception networks can extract richer features and avoid overfitting.

Notably, ResNet can achieve good network performance by increasing the depth of the network. Its main idea is that by using short-circuited links, the original information can be transmitted directly to the layers behind the network, protecting the integrity of the information and avoiding network degradation. Considering that the rice pest recognition task requires a balance between high accuracy and low complexity, we proposed a new model by improving ResNet18 [8] and MobileNetV2 [25]. The ResNet18 model was improved to accurately detect subtle information in images, while the MobileNetV2 model was enhanced to reduce the size of model parameters.

## 3. The Proposed Method

### 3.1. The Original Method

This section introduces our original method for classifying rice pests and diseases. One limitation of the BasicBlock in ResNet 18 is that the receptive field of its 3 × 3 convolution is too small for capturing rice pest and disease features in field environments. However, expanding the convolution size significantly increases the number of model parameters and FLOPs, resulting in a heavier network load and increased learning costs for the model. To expand the receptive field while maintaining a constant or reduced number of parameters and FLOPs, we replaced the 3 × 3 convolution with depthwise convolution. Additionally, to further increase the accuracy of the model, we wanted it to learn information about the same position of different channels, so we introduced the CBAM attention mechanism. CBAM is a potent attention mechanism that utilizes information from various channels and spaces. We named this improved initial model CBAMD-ResNet, and the code is summarized in Algorithm 1, where conv1 is a 7 × 7 depthwise convolution and conv2 is a 3 × 3 convolution. The trained CBAMD-ResNet model demonstrated a high level of accuracy, achieving a score of 93.07% on the test set.
**Algorithm 1:** CBAMD Block Forward**Require:** *x* (Input tensor)**Ensure:** *out* (Output tensor)1: **function** FORWARD(*x*)2: identity ← x3:      **if** downsample ≠ None **then**4:       *identity* ← downsample(*x*)5:      end if6:      *out* ← conv1(*x*)7:      *out* ← bn1(*out*)8:      *out* ← ReLU(*out*)9:      *out* ← conv2(*out*)10:    *out* ← bn2(*out*)11:    *out* ← ca(*out*) ·*out*12:    *out* ← sa(out)·out13:    *out* ← out + identity14:    *out* ← ReLU(*out*)15:    return *out*16: end function

### 3.2. The Proposed Classification Model

**Figure 1:**
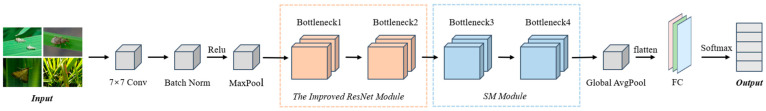
LMN Model Structure.

CBAMD-ResNet performed well on the test set but still needed further improvement in model size and accuracy, so we proposed an improved lightweight model, which is presented in this section. The overall structure is shown in Figure 1. We used ResNet18 as the base model, and the first two layers consist of an improved ResNet block layer. The improved ResNet block layer has a larger receptive field and a better ability to learn complex scale features and a better generalization ability than the traditional ResNet layer. To decrease the model size, we used the SM block layer for the final two layers of the model. The SM block’s design draws inspiration from the inverted bottleneck structure of MobileNetV2 and the channel shuffle technique of ShuffleNet, enhancing the model’s feature extraction capability while maintaining a lightweight architecture and reducing training costs. The following two sections will introduce these two block layers in detail.

### 3.3. The Improved ResNet18 Module

The advantage of traditional ResNet is that it solves the problem of network performance degradation using residual structures. Moreover, traditional ResNet alleviates the problem of gradient vanishing or gradient explosion by using short-circuiting links [8]. However, the 3 × 3 convolutional layer of ResNet18 extracts a limited target receptive field. It still has much room for improvement in the task of recognizing large-scale objects [26]. In addition, using a single-sized convolutional kernel makes it difficult to recognize multi-scale features.

**Figure 2:**
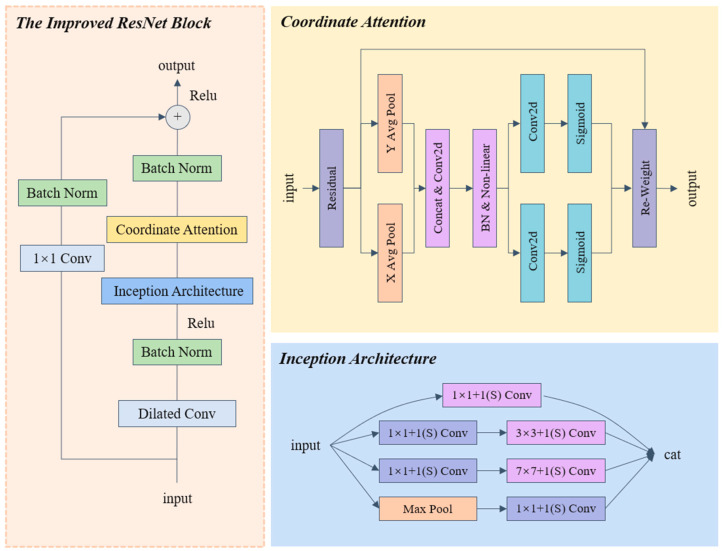
Structure of the improved ResNet block layer.

To solve the problem of the small receptive field extracted by traditional convolutional kernels, we improved the traditional ResNet18 model. Instead of the first 3 × 3 convolutional layer of ResNet18, we used dilated convolution [24]. This technique increases the size of the convolutional kernel, which can enhance the model’s receptive field. In order to learn the features at different scales, we introduced the inception convolution structure [7] to the second convolutional layer, which performs different convolution operations, such as 1 × 1, 3 × 3, and 7 × 7, and pooling operations on the input images in parallel. All the outputs were joined into a deep feature map. To maintain the original number of channels before convolution, the output channels of the convolution were reduced to one-quarter of their original value. In addition, the coordinate attention mechanism was introduced into the model to improve its sensitivity to channel information [23]. The coordinate attention mechanism can enhance the model’s ability to recognize critical regions in the input image by incorporating positional information into channel attention. This mechanism not only considers the relative importance of each feature channel but also integrates spatial location information, enabling the model to locate and focus on important regions more effectively. In complex scenes, this attention mechanism allows the model to better capture features associated with rice pests and diseases while minimizing the interference of irrelevant background information, ultimately improving classification accuracy. Figure 2 illustrates the structure of the improved ResNet model, and Algorithm 2 provides a summary of the code.
**Algorithm 2:** Improved ResNet Block Forward**Require:** *x* (Input tensor)**Ensure:** *out* (Output tensor)   1: **function** FORWARD(*x*)   2:      identity ← x   3:      *out* ← dilated_conv3 × 3(*x*)   4:      *out* ← bn1(*out*)   5:      *out* ← ReLU(*out*)   6:      *out* ← Inception(*out*)   7:      *out* ← coord_attention(*out*)   8:      *out* ← bn2(*out*)   9:      **if** downsample ≠ None **then**   10:       *identity* ← downsample(*x*)   11:       **if** identity_conv = None **then**   12:         identity_conv ← Conv2d(identity.size(1), out,size(1), kernel_size = 1, stride = 1, padding = 0, bias = False)   13:         identity_bn ← BatchNorm2d(out.size(1))   14:       end if   15:    *identity* ← identity_conv(*identity*)   16:    *identity* ← identity_bn(*identity*)   17:    end if   18:    out ← out + identity   19:    *out* ← ReLU(*out*)   20:    **return** *out*   21: end function

### 3.4. SM Module

The SM block was introduced mainly to improve the model’s performance and reduce the parameters and FLOPs of the model, which reduces the computational cost by incorporating it with deep convolution.

**Figure 3:**
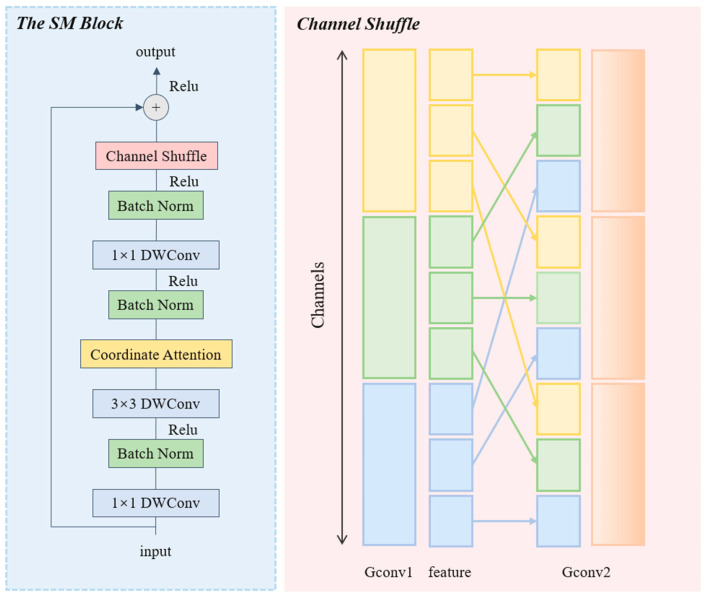
Structure of the SM block layer.

To address the issue of ResNet18’s large parameters, we utilized the inverse bottleneck structure of MobileNetV2 [25]. When designing the convolution, we replaced all the 1 × 1 and 3 × 3 conventional convolutions with depthwise convolution. Traditional convolution mixes all channel information to generate a new output channel, while mobileNetV2’s depthwise convolution separates this process by performing convolution operations on each input channel individually. This significantly minimizes the computational cost of the model. However, since depthwise convolution cannot take into account the relationships between different channels, we specifically introduced the channel shuffle operation to address this limitation. This idea was inspired by ShuffleNet, as proposed by Zhang et al. [27]. This operation disrupts the order of the original feature map channels and re-groups them so that the group convolutional layer can learn the complementary information between different feature channels, which facilitates the fusion of information across channels. Additionally, channel shuffle is a simple reorganization operation that is extremely lightweight and does not impose significant computational costs while preserving the efficiency of depthwise convolution in MobileNet to the greatest extent possible. Similarly, we also introduced the coordinate attention mechanism [23] in the SM block to increase the sensitivity of the model to channel information. Figure 3 illustrates the structure of the SM block layer, while Algorithm 3 provides a summary of the code.
**Algorithm 3:** SM Block Forward**Require:** *x* (Input tensor)**Ensure:** *out* (Output tensor)   1:   **function** FORWARD(*x*)   2:     *identity* ← *x*   3:     *out* ← Depthwise_conv1(*x*)   4:     *out* ← bn1(*out*)   5:     *out* ← ReLU(*out*)   6:     *out* ← Depthwise_conv2(*out*)   7:     *out* ← coord_attention(*out*)   8:     *out* ← bn2(*out*)   9:     *out* ← Depthwise_conv3(*out*)   10:   *out* ← bn3(*out*)   11:   *out* ← channel_shuffle(*out*)   12:   **if** downsample ≠ None **then**   13:     *identity* ← downsample(*identity*)   14:   end if   15:   *out* ← *out* + *identity*   16:   *out* ← ReLU(*out*)   17:   **return** *out*   18: end function

## 4. Experiments

### 4.1. Dataset

Given the fact that the health status of crops is affected by a variety of factors, including pests and diseases, the construction of a dataset that includes both pests and diseases can simplify the data annotation process and enable the model to more comprehensively identify the condition of the crop. Therefore, seven pest types that are highly damaging and have a wide range of impacts were selected and compiled in a unified dataset. The selected pest types include the white-backed planthopper, the rice water weevil, and the rice leaf-roller. The selected diseases include rice blast, rice tungro spherical virus, helminthosporium leaf spot, and bacterial leaf. The distinct characteristics of these pests and diseases can, to some extent, reflect the traits of various types of pests and diseases found in natural organisms, making them ideal representatives for training pest and disease recognition models.

The image data for this study were mainly collected from publicly available datasets on the Internet. The rice pest dataset was sourced from the IP102 agricultural pest dataset constructed by Wu et al. [28], and the rice disease dataset was obtained from a public dataset on Kaggle called Philippines Rice Diseases. Finally, the fused dataset was formed through integration and classification. However, the dataset also includes some misclassifications, irrelevant information, and varying image quality. To minimize bias and ensure the accuracy and representativeness of the data we have done the following work:

(1) The fused dataset was expanded to seven categories by retrieving the rice pests and diseases images on the network and using web crawling techniques.
(2) We manually inspected the dataset by reviewing information related to agricultural pests and querying the Image Dataset for Agricultural Diseases and Pests (IDADP) dataset. Under the guidance of agricultural experts, we removed misclassified, duplicate, and low-quality images.

**Figure 4:**
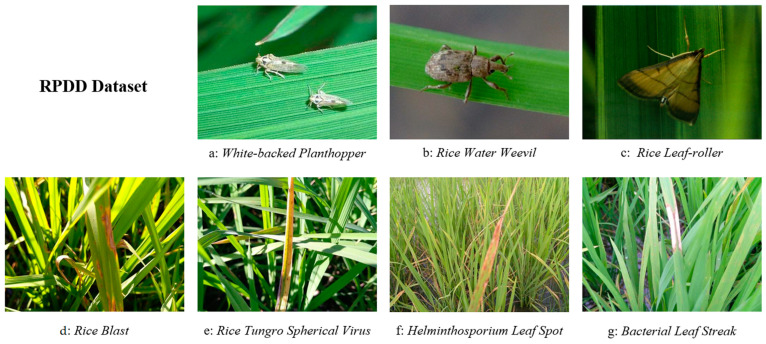
Example samples from the RPDD.

**Figure 5:**
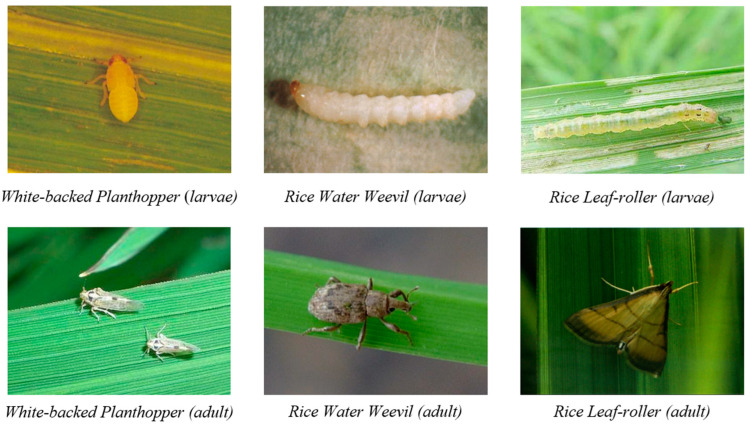
Example samples of larvae and adult pests.

The RPDD includes seven distinct categories of rice diseases and insect pests, offering a diverse range of images captured in various environmental conditions. The images capture various angles and postures of the diseases and pests, including different growth stages of the pests (both larvae and adults). Most were taken in natural field environments, using a range of devices under different lighting conditions, as illustrated in Figure 4 and Figure 5. In comparison to other publicly available datasets, the RPDD dataset provides higher-quality images, reducing the risk of introducing training biases that may arise from an overrepresentation of specific pests or diseases. This enhances the model’s generalization capacity, particularly in the context of large-scale datasets for future training.

### 4.2. Data Augmentation

To alleviate the problem of limited images and imbalanced sample distribution, as well as to prevent the model from overfitting, we employ data augmentation strategies to extend the dataset. By transforming, editing, and modifying the data, the sample features can be enriched and the training bias can be reduced. To enhance the model’s capacity for generalization, two data fusion methods, namely MixUp and CutMix, were employed in the course of the experiments. A detailed description of these data augmentation methods is given below.

#### 4.2.1. Traditional Data Augmentation

The traditional methods for expanding the dataset involve geometric transformations and pixel transformations [29]. These conventional approaches effectively increase the sample size and improve the performance of deep learning networks. Data augmentation considers the proportional distribution of images across rice disease and pest categories. Greater augmentation is applied to categories with fewer samples, while categories with more samples are subjected to lesser augmentation. Based on this strategy, a more balanced sample distribution is achieved.

**Figure 6:**
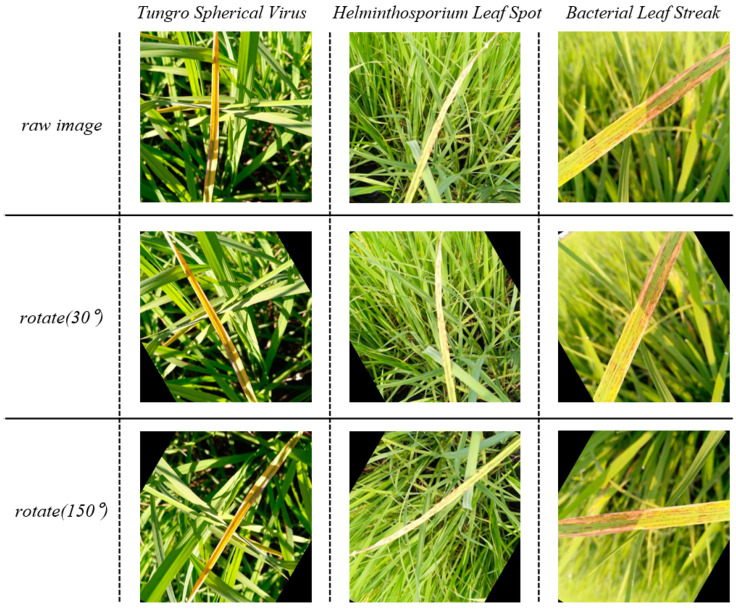
Image samples of geometric transformation results.

Geometric transformations commonly include translation, rotation, cropping, mirroring, and scaling. The geometric transformation method we employed is rotation. Taking tungro spherical virus, helminthosporium leaf spot, and bacterial leaf streak as examples, the results are shown in Figure 6.

**Figure 7:**
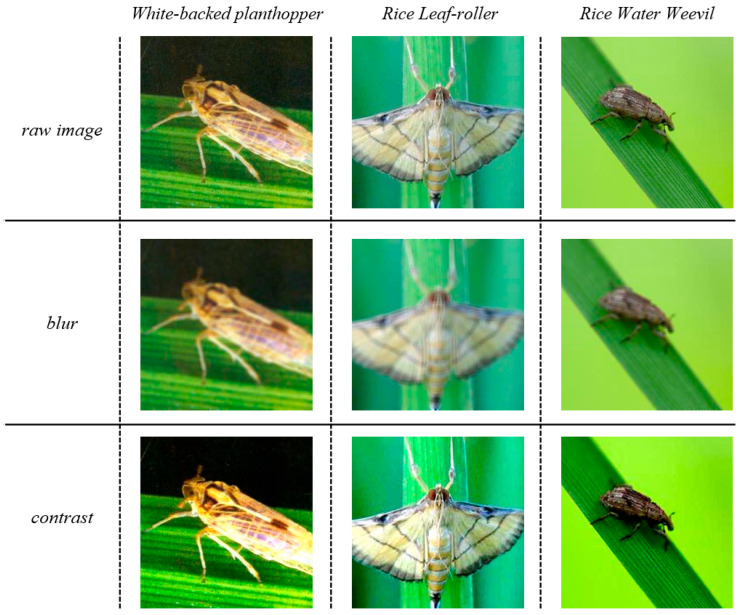
Image samples of pixel transformation results.

Geometric transformation focuses on changing the geometric and shape characteristics of an image. The model’s ability to perceive images from different angles can be improved by using geometric transformation methods. In addition to taking into account the changing shape of the image, our model should also adapt to the visual characteristics of the image that change due to different devices, photographers, environments, etc. Pixel transformations change an image’s visual properties, including brightness, contrast, and color, by modifying pixel values. Pixel transformations encompass operations such as adding salt-and-pepper noise, applying Gaussian blur, adjusting brightness, saturation, and white balance, and histogram equalization. The transformation methods we used include applying Gaussian blur and adjusting contrast. Taking as examples the white-backed planthopper, rice leaf-roller, and rice water weevil, the results obtained are shown in Figure 7.

#### 4.2.2. Data Fusion Methods: MixUp and CutMix

Due to the limited variations among different images of the same disease, the model may learn irrelevant information. This leads to overfitting and poor generalization performance. To address these issues, we incorporated image fusion and stitching techniques to generate mixed samples from two or more data samples. Representative approaches in this regard include Mosaic [30], MixUp [31], and CutMix [32]. The purpose of these design modifications is to enhance the model’s robustness and decrease its sensitivity. We employed MixUp and CutMix to create two distinct augmented datasets. We selected the most suitable method through experiments and evaluations of the augmented datasets.

The core idea of MixUp is to randomly select two image samples from each batch for feature mixing and superposition. Then, this strategy controls the mixing coefficient through beta distribution to generate new sample data [31]. The entire process is expressed by the formula

(1) λ∼Beta(α,α),

(2) $$\tilde{X}=\lambda X1+(1-\lambda )X2,$$

(3) $$\tilde{Y}=\lambda Y1+(1-\lambda )Y2\;$$

where λ follows a *Beta* distribution with both parameters set to α. *X*1 and *X*2 represent two randomly selected input samples, while *Y*1 and *Y*2 denote the corresponding one-hot encoded labels. $\tilde{X}$ and $\tilde{Y}$ denote the linearly superimposed features and labels, respectively.

CutMix involves removing a portion of the image and filling it with regional pixel values using the rest of the data in the training set [32]. The results of the classification are then distributed based on a certain proportion. CutMix data augmentation uses a region-based dropout strategy to improve the model’s focus on the essential parts of the target. The dropout strategy enhances the network’s generalization and the ability to locate key regions accurately. *Xa* and *Xb* are two training samples with different labels. The label values *Ya* and *Yb* correspond to each other. The CutMix network generates new training samples $\tilde{X}$ and their corresponding labels $\tilde{Y}$ through this process. The whole process is represented by the following equation:

(4) $$\tilde{X}=\left(1-M\right)⊙Xa+M⊙Xb,$$

(5) $$\tilde{Y}=\lambda ⊙Ya+(1-\lambda )⊙Yb,$$

The symbol *M*∈{0,1}^W × H represents a binary mask that indicates the cropping and filling regions. The operation ⊙ denotes element-wise multiplication. The parameter λ follows a *Beta* distribution, which can be denoted as λ ∼ *Beta*(α, α).

CutMix combines two training samples from a single batch with different types by blending their labels and image contents. This approach expands the dataset and enhances the performance of the network during training.

### 4.3. Data Partitioning

We randomly reshuffled the order of the data to avoid bias in the training results and to improve the robustness of the model. To improve the precision of model parameters and prevent overfitting, the dataset was randomly divided into training, validation, and test sets according to a 6:2:2 ratio [33], as shown in Table 1. The training set shown in Table 1 contains the images from the original training set as well as the augmented images, while the original training set contains only raw images. Each dataset includes images representing different growth stages of the pest and images under different light conditions.

### 4.4. Evaluation Metrics

Various metrics are used to evaluate the model’s performance, such as accuracy, loss value, F1-Score, model parameter size, and FLOPs. The definition and calculation of some important metrics are described below. *TP* (True Positive) represents the number of positive instances that the model correctly identified as positive; *TN* (True Negative) represents the number of negative instances that the model correctly identified as negative; *FP* (False Positive) represents the number of negative instances that the model incorrectly identified as positive; *FN* (False Negative) represents the number of positive instances that the model incorrectly identified as negative [34].

*Accuracy* assesses the classification precision of a model. It is calculated by taking the number of samples that were correctly predicted and dividing it by the total number of samples. The accuracy of the model is represented as follows:

(6) $$Accuracy=\frac{TP+TN}{TP+TN+FP+FN}\;,$$

The concept of *precision* in this context refers to the closeness between the predicted value and the true value. It is computed as shown in the following equation:

(7) $$Precision=\frac{TP}{TP+FP}\;,$$

*Recall* is the probability of correctly predicting a positive sample. It can be expressed as the ratio between the true positive values predicted and all positive values, as follows:

(8) $$Recall=\frac{TP}{TP+FN}\;,$$

The *F1*-Score is an evaluation metric for multi-class classification. The *F1*-Score considers both precision and recall. It can be seen as a weighted average of the model’s precision and recall. *F1*-Score is represented as follows:

(9) $$F1=2\times \frac{Precision\times Recall}{Precision+Recall}\;,$$

### 4.5. Experimental Environment and Parameter Settings

The experiment’s setup included the use of the following: Windows 11 operating system, 12th generation Intel(R) Core(TM) i9 12900KF processor, GeForce RTX 3080 graphics card with 12 GB of video memory, and 64 GB of RAM. We used PyTorch based on Python 3.9.12 to train the model. Table 2 lists the information about the specific experimental parameters we used in training the model.

### 4.6. Experiment to Select MixUp and CutMix

To evaluate the two data enhancement methods MixUp and CutMix, we proposed a strategy for evaluating these data enhancement methods. The rice pest and disease dataset was augmented by MixUp and CutMix data. Thus, we obtained two different training sets. The ResNet18 was chosen as the learning network. We conducted experiments using the same test set and obtained experimental results for the ResNet18 based on the two training sets. The dataset with the higher accuracy rate was selected as the rice pest and disease augmented dataset.

As the results shown in Table 3 demonstrate, we found that the MixUp data enhancement method has a negative impact on the training results of the ResNet18 network with respect to the RPDD. This could be due to the fact that mixed samples in MixUp are too large. These samples deviate from the actual data distribution, resulting in difficulties in training the model. On the contrary, the accuracy when using the CutMix data enhancement method increased by 0.22 percentage points. Furthermore, the F1-Score of the network using CutMix was also higher, and the loss value was lower. It can be seen that using CutMix effectively enhances the training performance of the model. Therefore, we adopted CutMix as the data enhancement method to produce the enhanced rice pest and disease dataset.

We further investigated the differences in the performance of these two data enhancement techniques under different network architectures. As seen in the experimental results shown in Table 4, we found that for ResNet and InceptionNeXt, data enhancement using CutMix outperformed Mixup. However, for ConvNeXt, ParCNetV2, MobileNetV2, and ShuffleNet v2, Mixup outperformed CutMix. The possible reasons are the differences in structural complexity and the feature extraction methods of these networks.

CutMix and Mixup are both data enhancement techniques used to enhance the robustness of image classification models. Mixup works by linearly interpolating two random images, resulting in a new image with more ambiguous features. This may reduce the model’s sensitivity to boundaries and local details. Therefore, in tasks where detailed features need to be recognized, using Mixup can make model training more challenging. CutMix creates a new image by pasting part of one image onto another, retaining not only the original information of both images but also the key structural features of the objects. This facilitates the model’s ability to learn local features with greater precision. The difference in how they handle detailed features leads to CutMix outperforming Mixup when training with the ResNet network. Since ResNet uses residual blocks to extract and transfer features layer by layer, the preservation of local features helps ResNet develop a deeper understanding of the image at various levels, which enhances its performance when using CutMix in this architecture. InceptionNeXt employs the inception style, which focuses on feature extraction at multiple scales, and thus also adapts well to CutMix data enhancement techniques. We have thus fused the inception module into our improved ResNet network.

ConvNeXt and ParCNetV2, which use large convolution kernels to enhance the receptive field, are more suitable for large-scale feature extraction. This is why images generated by Mixup are more favorable for ConvNeXt and ParCNetV2 compared to CutMix. MobileNetV2 and ShuffleNet v2 are lightweight networks with fewer parameters, making them less capable of learning fine-grained features compared to more complex convolutional networks like ResNet. As a result, they struggle to effectively capture local details and boundary information, sometimes even mistaking clear boundaries for noise. In contrast, the smooth samples generated by Mixup are more conducive to the training of these networks.

### 4.7. Comparison of Different Models

We compared the network performance of the RPDD under ten different models: ResNet18, ResNet34, ResNe50 [8], ConvNeXt-tiny [35], InceptionNeXt-small, InceptionNeXt-tiny [36], ParCNetV2 [37], MobileNetV2 [25], ShuffleNet-v2 1×, ShuffleNet-v2 0.5× [38], and the proposed model LMN. The results are presented in Table 5.

**Figure 8:**
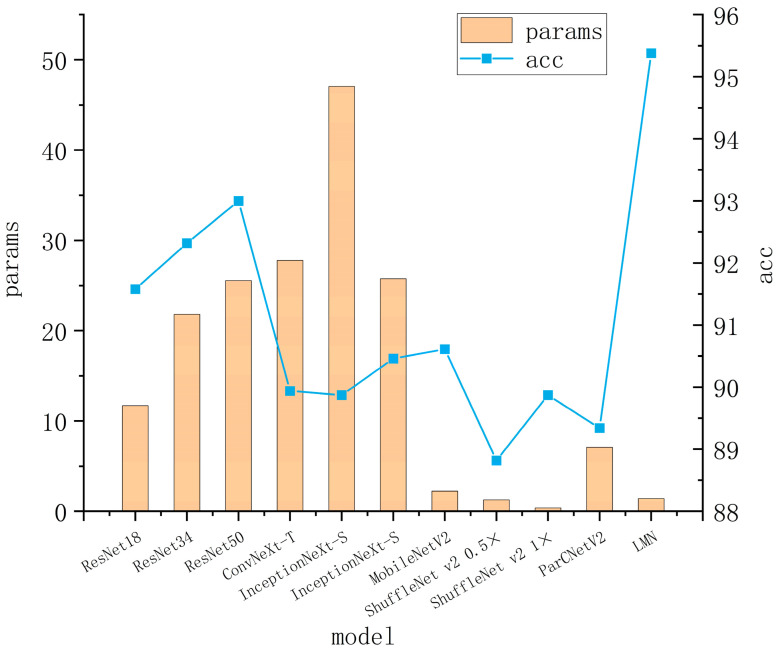
Comparison of different model parameters and accuracy results.

Based on the analysis shown in Table 5, our proposed model achieves the highest accuracy among all tested models with a score of 95.38%, which is 3.8% higher than the baseline structure. Compared with ResNet34, ResNet50, ConvNeXt-tiny, InceptionNeXt_small, InceptionNeXt_tiny, ParCNetV2, MobileNetV2, ShuffleNet v2 1×, and ShuffleNet v2 0.5×, the accuracy of our model is improved by 3.06%, 2.38%, 5.44%, 5.51%, 4.92%, 7.01%, 4.77%, 5.51%, 6.56%, respectively. In terms of loss value, our model achieves the lowest loss value compared to the others. This indicates that our model has better fitting results. The F1-Score of our model is 94.50%, which implies that the network model we constructed has the highest stability and generalization ability compared with other classification models. In terms of model parameters, our model significantly reduces the parameters of the base models by 88.02%. Compared to the popular lightweight models MobileNetV2, ShuffleNet v2 1×, and ShuffleNet v2 0.5×, our model is not only smaller, but also greatly improves the recognition accuracy. It is worth noting that the proposed LMN model significantly outperforms other models in terms of model size and overall accuracy, as illustrated in Figure 8.

On the whole, the results show that our model improves the recognition performance, and its complexity is also desirable. Therefore, our model is more suitable for deployment on the website.

**Figure 9:**
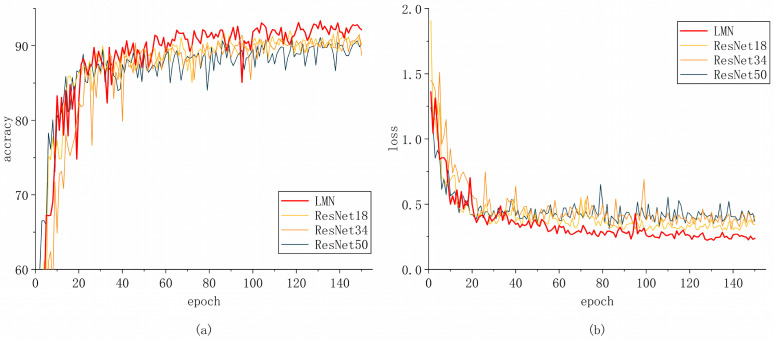
Comparison of LMN with ResNet18, ResNet34, and ResNet50; (**a**) accuracy and (**b**) loss.

**Figure 10:**
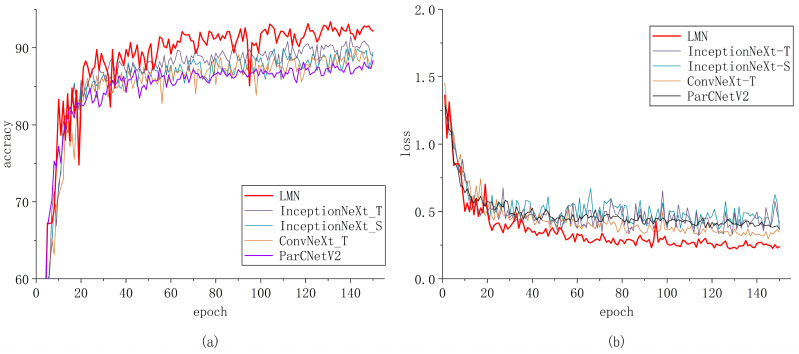
Comparison of LMN with ConvNeXt-tiny, InceptionNeXt_small, InceptionNeXt_tiny, and ParCNetV2; (**a**) accuracy and (**b**) loss.

**Figure 11:**
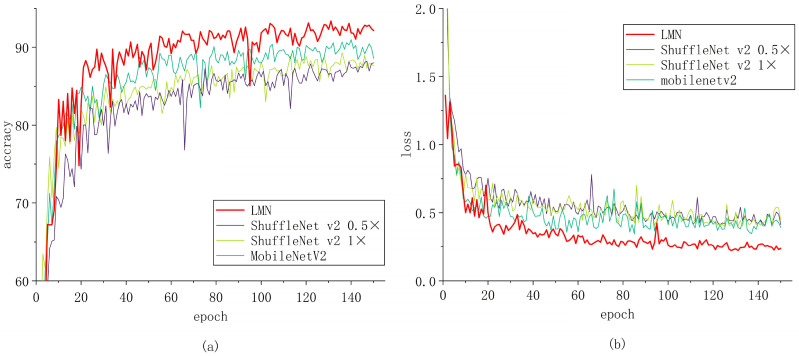
Comparison of LMN with MobileNetV2, ShuffleNet v2 1×, and ShuffleNet v2 0.5×; (**a**) accuracy and (**b**) loss.

We observed the convergence of our model and the other models. The results are shown in Figure 9, Figure 10 and Figure 11. This visualization can help us to better understand the improvement achieved in network learning performance.

We trained the model for 150 epochs. Initially, the model converged at a faster rate, characterized by larger gradients and more significant parameter updates. However, as the model gradually fitted the training data, the convergence speed began to slow down after approximately 20 epochs, leading to a stabilization in the decline of the loss function. The refined parameter updates were instrumental in ensuring the model’s convergence and in effectively mitigating the risk of overfitting.

Our model significantly outperformed lightweight models (MobileNetV2, ShuffleNet V2 1×, and ShuffleNet V2 0.5×), image classification models (ConvNeXt-tiny, InceptionNeXt_small, InceptionNeXt_tiny, and ParCNetV2), and the traditional ResNet family of models (ResNet18, ResNet34, and ResNet50). During this stage, the convergence of the models slowed further, and the more fine-grained weight updates facilitated a better understanding of the subtle and complex features of the models.

In summary, our proposed network model shows good performance in the task of recognizing rice pests and diseases.

### 4.8. Ablation Experiment

We conducted ablation experiments based on the base network ResNet18 to evaluate the impact of the improved ResNet module and the SM module on the model’s performance. The results of the experiment are presented in Table 6. The accuracy of the LMN model reached 95.38%, and the F1-Score reached 95.50%. The number of parameters was 1.4 M. We found that the coordinate attention mechanism improved the accuracy of the model by 1.49%. The coordinate attention mechanism reduced the loss value, but resulted in a small increase in the model parameters by 0.64 M. This is due to the fact that we introduced more parameters to make the model better handle the structure of the data during the feature capture process.

The improved ResNet module improves accuracy by 0.3%, and it effectively reduces the model parameters by 0.16 M and computation by 0.42. It is notable that our proposed *SM* module drastically reduces the parameters. The model size after introducing the SM model is only 7.87% of the original model, and its accuracy is significantly improved by 2.01% over the base model. There are also different degrees of improvements in loss and FLOPs. The above results show that our improved ResNet module, the SM module, and the introduced coordinate attention mechanism have enhanced the performance of the rice pest and disease recognition model to different degrees.

To achieve the optimal recognition performance of the convolutional model, we conducted a series of comparative experiments by arranging and combining the improved convolutional layers. The experimental results are presented in Table 7, where ‘R’ denotes the improved *ResNet18* module and ‘S’ denotes the proposed SM module. We found that R_1_R_2_S_3_S_4_ outperformed the other combinations with an accuracy of 93.89%. Therefore, we chose the structure of R_1_R_2_S_3_S_4_ for further experiments.

### 4.9. Comparison of Different Attention Mechanism Models

The choice of attention mechanism has a notable effect on the outcomes of the model when applied to image classification and target detection tasks. The channel attention mechanism can minimize the parameter size of the model to avoid introducing large overheads. Meanwhile, the channel attention mechanism still has sufficient accuracy. Hence, we have chosen three channel attention mechanisms applicable to lightweight models, i.e., *SE*, *ECA*, and *CA* [21,22,23]. The experimental results are shown in Table 8.

According to Table 8, the accuracy of the model is improved by 0.30%, 0.07%, and 1.49% after introducing *ECA*, *SE*, and *CA* attention mechanisms, respectively. It can be seen that the *CA* attention mechanism better improves the model performance compared to other attention mechanisms. It indicates that the coordinate attention mechanism is more suitable for the rice pest recognition task. This may be because the *CA* attention mechanism mainly focuses on the relationship between spatial coordinates rather than feature channels. In the rice pest and disease classification task, there may be some important features related to spatial locations, such as insect body parts or specific structures of plants. The *CA* attention mechanism can capture the spatial relationships between diverse features within an image and extract more representative features, thus improving the classification performance. Thus, we choose the *CA* attention mechanism to optimize the model.

### 4.10. Confusion Matrix

**Figure 12:**
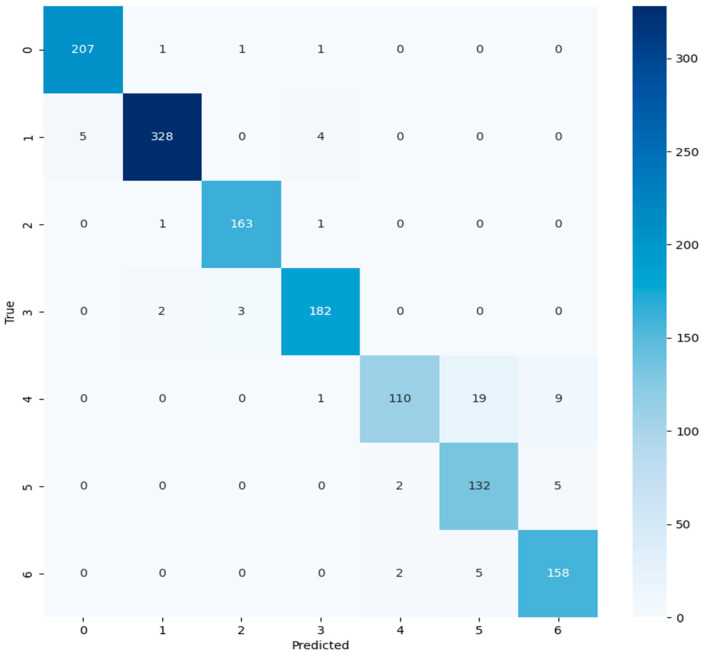
Visualized result of confusion matrix.

The confusion matrix can visualize the model’s classification results and show the recognition effect intuitively. The confusion matrix was used to analyze the experimental results to further evaluate the model’s performance. The results of the LMN model in to classifying the test set data are illustrated in Figure 12. The results show that three images of label 0 were incorrectly classified. Nine images of label 1 were incorrectly classified. Two images of label 2 were incorrectly classified. Five images of label 3 were incorrectly classified. Twenty-nine images of label 4 were incorrectly classified. Seven images of label 5 were incorrectly classified. Seven images of label 6 were incorrectly classified. The analysis shows that the model recognizes label 2 best. Labels 4 and 5 had a higher probability of being confused. The results indicate that the LMN model is effective in identifying rice pests and diseases.

### 4.11. Web Deployment

**Figure 13:**
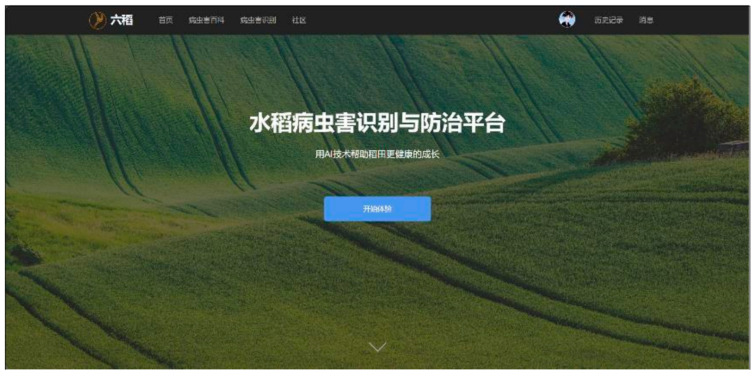
Platform homepage. The home page of a rice pest and disease identification and control platform.

**Figure 14:**
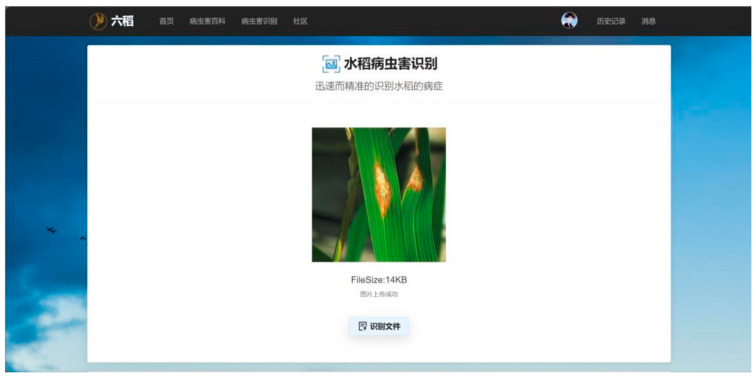
Image upload page. This page is the interface of the rice pest and disease identification system where the user uploads a picture of the rice disease for identification.

**Figure 15:**
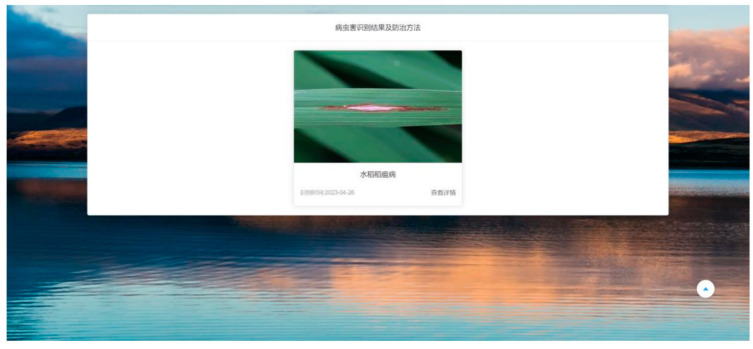
Rice disease identification results page. This page shows the identification results, identifying the disease as ‘rice blast’, and provides an option to search for detailed information.

We deployed our LMN model on our website and developed a well functioning rice pest and disease recognition platform, where users can upload pictures of rice pests and diseases. Users can view the control methods for these types of pests and diseases after successful recognition. The platform’s homepage is shown in Figure 13, the image upload page is shown in Figure 14, and the recognition result page is shown in Figure 15. We hope that this application will alleviate the burden on agricultural workers and researchers by allowing them to take pictures of diseases and upload them for diagnosis via smartphones. This enables remote disease detection and preliminary assessments without waiting for expert diagnosis, so that the occurrence and spread of pests and diseases can be prevented and controlled in the shortest possible time.

To promote the application, it is essential to gather further valuable user feedback to evaluate the effectiveness of the system and identify areas for improvement. The simplicity of the user interface and its ease of use are of primary concern. Furthermore, given the potential for unstable network connections in the field environment, future attention will be paid to the offline operation function of the application. Also, when a high volume of users accesses the application concurrently, it may encounter performance issues, such as slow response times or unexpected disruptions in service. We need to improve the concurrent processing capability of the application to increase its performance.

Since we only labelled the data and trained the model for seven types of rice pests and diseases, our model may get incorrect classification results if it needs to identify other types of rice pests and diseases in practical use. Therefore, we still need to obtain more types of pest and disease data to enrich the training set of the model. We will also introduce migration learning techniques into our model for the recognition of more types of pests and diseases.

In addition, the development of Internet of Things (IoT) technology has enabled smart agriculture. By integrating sensors, drones, and automated equipment with our recognition systems and intelligent decision support, precise crop monitoring and management can be achieved. The recognition system can promptly detect early symptoms of pests and diseases, automating part of the diagnostic process. This helps to effectively prevent disease spread, protect crop health and yields, and enhance the economic efficiency of agricultural production.

## 5. Discussion

We compared our model to research in the field of pest and disease identification tasks. Our model employs more advanced lightweight techniques compared to existing models. By introducing the inception module, our model uses different sizes of convolutional kernels in parallel to extract information at different scales. This allows the model to capture multiple features of the input image. In addition, we introduced a lightweight attention mechanism in the model, which does not significantly increase the parameter size. It can effectively improve the model’s performance without increasing the consumption of computational resources. The ECA-ConvNeXt model proposed by Wang et al. [39] was able to identify seven types of diseased leaves with an accuracy of 94.8%. In contrast, our model is capable of identifying not only diseases but also pests, as well as improving the accuracy by 0.6%. This improvement is mainly attributed to the usage of an advanced multi-scale feature extraction module. It can effectively capture image features at different scales, offering significant advantages in dealing with complex and diverse pest and disease images. In addition, our dataset has a higher background complexity compared to the model proposed by Shreya Ghosal et al. [40]. By incorporating the CA attention mechanism, our model can better capture critical regions within images, ultimately resulting in a 2.9% higher accuracy than the model proposed in their study. Furthermore, our model identifies a wider variety of pests and diseases compared to the model presented by Wang et al. [41]. It also uses data augmentation techniques to reduce the risk of overfitting and significantly improve generalization, resulting in a 0.7% increase in accuracy.

There are still some shortcomings in our study. As mentioned in the previous section, pests and diseases do not exist independently in the agricultural environment. When multiple pests or diseases appear in the same image at the same time, our model may misidentify or identify them incompletely. To address these complex combinations, we are considering a multi-label classification technique. However, obtaining multi-label datasets suitable for pest and disease identification is challenging. Furthermore, due to the small number of parameters in the LMN model, the model may be insufficiently trained, reducing the model’s performance. Additionally, increasing the output dimensions implies higher computational resource requirements and longer processing times.

A pest and disease dataset is typically derived from a specific geographical region. However, climatic and soil conditions in different regions may lead to differences in the performance of pests and diseases, which in turn makes it difficult for the model to adapt effectively in different environments. Therefore, further research and enhancements are necessary to enhance the model’s adaptability and recognition accuracy across different environments.

In the future, we will do more research in three-dimensional(3D) image recognition and object detection based on pest and disease data. 3D model recognition technology is capable of detecting and diagnosing pests and diseases with greater precision, particularly in circumstances where two-dimensional(2D) images lack sufficient detail. For example, pests and diseases can cause subtle structural changes in crops. These may include depressions, bumps, or leaf curl deformations on the crop surface. Such features are difficult to detect accurately using traditional 2D image recognition models. However, 3D recognition techniques can capture these surface anomalies more clearly. This leads to more accurate detection results. Nevertheless, those migrating to 3D recognition models are still confronted with many challenges and potential difficulties. 

Firstly, 3D data capture and labeling is very challenging. It is more difficult to acquire high-quality 3D data, and the labelling process is more complex than for 2D images. In addition, there is a significant difference in the representation of 2D and 3D images. 2D images are pixel matrices, while 3D data can be in the form of 3D meshes, point clouds, etc. Therefore, our current method of extracting spatial features based on 2D convolutional layers cannot be directly applied to 3D data, which requires redesigning the convolutional kernel and the pooling method for different forms of 3D data. In addition, the increased input dimension of 3D models also puts higher demands on computational resources, which requires the consideration of how to efficiently utilize the available computational resources to keep the models lightweight and low-latency.

Despite the many challenges, migrating models to 3D identification is still feasible. Encouragingly, our laboratory has abundant agricultural resources and advanced equipment that can be used for 3D data collection and the modeling of crop pests and diseases, thus making future research possible. We are also looking at how the existing model can be adapted for 3D identification tasks. For example, the ResNet network structure we use has good adaptability, and the residual connection mechanism can be applied in 3D convolutional models to avoid the gradient vanishing problem. We can replace the 2D convolution in the ResNet network with 3D convolution to enable the model to extract features in 3D space. In addition, many researchers have now proposed lightweight 3D recognition network models, such as LightNet and PointNet [42,43], etc. We believe that we can draw on the useful experience of these networks to reduce the parameter size and computational consumption of the models.

## 6. Conclusions

Our study focuses on two main aspects.

Firstly, we constructed a dataset through image augmentation techniques to address the problem of the lack of comprehensive public datasets for rice pests and diseases. We selected representative types of pests and diseases in nature, which allows our model to have good generalization.

Secondly, we proposed a lightweight LMN model based on ResNet, which effectively improves the performance of the model in the task of identifying rice pests and diseases by introducing operations such as the coordinate attention mechanism, the inception module, and channel shuffling. The comprehensive performance of our model outperformed other convolutional networks.

The LMN model has a wide range of applications given its lightweight design. Its application in agricultural production and research can reduce the workload of people and contribute to the improvement of food production and economic benefits.

## Figures and Tables

**Table 1:** Partition of RPDD.

Category	Test Set	Training Set	Validation Set	Original Training Set	Total
White-backed Planthopper	139	2496	139	416	2774
Rice Water Weevil	139	2490	138	415	2767
Rice Leaf-roller	165	2970	165	495	3300
Rice Blast	337	2018	337	1009	2692
Rice Tungro Spherical Virus	210	3150	210	630	3570
Helminthosporium Leaf Spot	187	2800	187	560	3174
Bacterial Leaf Streak	165	2465	165	493	2795

**Table 2:** Experimental parameter information.

Parameter	Value or Name
Input image size	224 × 224
Batch size	64
Training epochs	150
Learning rate	0.001
Loss function	CrossEntropyLoss
Optimizer	Adam

**Table 3:** Results using different data augmentation methods.

Data Augmentation Method	Accuracy/%	Loss	F1-Score/%
ResNet18(BaseLine)	91.36	0.5379	90.14
ResNet18 + CutMix	91.58	0.2941	90.08
ResNet18 + MixUp	90.01	0.3571	88.84

**Table 4:** Accuracy comparison of different models under CutMix and Mixup data augmentation.

Model	CutMix	MixUp
ResNet18	91.58%	90.01%
ResNet34	92.32%	90.69%
ResNet50	93%	89.42%
ConvNeXt-T	89.94%	91.28%
InceptionNeXt-S	89.87%	89.49%
InceptionNeXt-T	90.46%	89.27%
ParCNetV2	88.37%	90.76%
MobileNetV2	90.61%	92.10%
ShuffleNet v2 1×	89.87%	89.94%
ShuffleNet v2 0.5×	88.82%	90.61%

**Table 5:** Comparison of experimental results for different models.

Model	Accuracy/%	Loss	F1-Score	Params	FLOPs
ResNet18	91.58	0.2941	0.9008	11.69	3.65
ResNet34	92.32	0.3361	0.9116	21.8	7.36
ResNet50	93	0.2792	0.9188	25.56	8.27
ConvNeXt-T	89.94	0.299	0.8871	28.57	8.91
InceptionNeXt-S	89.87	0.4324	0.8862	47.07	16.74
InceptionNeXt-T	90.46	0.4959	0.8927	25.76	8.4
ParCNetV2	88.37	0.37	0.8679	7.1	3.11
MobileNetV2	90.61	0.396	0.8946	3.5	0.65
ShuffleNet v2 1×	89.87	0.3325	0.8885	2.28	0.31
ShuffleNet v2 0.5×	88.82	0.4173	0.8724	1.37	0.09
**LMN**	**95.38**	**0.1779**	**0.945**	**1.4**	**1.65**

**Table 6:** Ablation experiment results of different models.

	Accuracy/%	Loss	F1-Score	Params	FLOPs
ImpRes + SM + CA (LMN)	95.38	0.1779	0.945	1.4	1.65
ImpRes + SM	93.89	0.2214	0.9279	0.76	1.63
OriRes + SM	93.59	0.2176	0.9241	0.92	2.05
OriResNet18	91.58	0.2941	0.9008	11.69	3.65

**Table 7:** Experimental results of network layer restructuring.

Number	Model	Accuracy/%	Loss	F1-Score	Params	FLOPs
1	R_1_R_2_R_3_R_4_	92.55	0.273	0.9126	1.4	1.65
2	R_1_R_2_R_3_S_4_	92.55	0.2425	0.9126	8.48	2.83
3	R_1_R_2_S_3_R_4_	92.77	0.263	0.9154	2.3	2.22
4	R_1_R_2_S_3_S_4_	93.89	0.2214	0.9279	6.95	2.23
5	R_1_S_2_R_3_R_4_	93.00	0.2486	0.917	0.76	1.63
6	R_1_S_2_R_3_S_4_	64.68	1.1928	0.6494	8.1	2.24
7	R_1_S_2_S_3_R_4_	93.89	0.2031	0.9266	1.92	3.58
8	R_1_S_2_S_3_S_4_	91.28	0.287	0.9006	0.38	1.04
9	S_1_R_2_R_3_R_4_	91.43	0.3614	0.902	8.38	2.15
10	S_1_R_2_R_3_S_4_	78.91	0.6429	0.7963	2.2	1.55
11	S_1_R_2_S_3_R_4_	90.61	0.3058	0.8908	6.85	0.64
12	S_1_R_2_S_3_S_4_	93.29	0.2483	0.9188	0.66	0.44
13	S_1_S_2_R_3_R_4_	89.42	0.3346	0.8797	8	1.53
14	S_1_S_2_R_3_S_4_	88.67	0.3815	0.8703	1.82	0.45
15	S_1_S_2_S_3_R_4_	90.54	0.3579	0.889	6.47	0.91
16	S_1_S_2_S_3_S_4_	59.02	1.1451	0.545	0.28	0.3

**Table 8:** Comparison results of different channel attention mechanisms.

	Accuracy/%	Loss	F1-Score
the improved model	93.89	0.2214	0.9279
the improved model + *ECA*	94.19	0.2550	0.9303
the improved model + *SE*	93.96	0.2258	0.9272
the improved model + *CA*	95.38	0.1779	0.9450

## Data Availability

The data and source codes that support the findings of this study are available from the corresponding authors, upon reasonable request.

## References

[1] Sharif M., Butt M., Anjum F., Khan S.H. (2014). Rice Bran: A Novel Functional Ingredient. Crit. Rev. Food Sci. Nutr..

[2] Plant Health and Climate Change. https://www.fao.org/3/cb3764en/cb3764en.pdf.

[3] Bren d’Amour C., Reitsma F., Baiocchi G., Barthel S., Güneralp B., Erb K.-H., Haberl H., Creutzig F., Seto K. (2017). Future Urban Land Expansion and Implications for Global Croplands. Proc. Natl. Acad. Sci. USA.

[4] Xiao Z., Yin K., Geng L., Wu J., Zhang F., Liu Y. (2022). Pest Identification via Hyperspectral Image and Deep Learning. Signal Image Video Process.

[5] Ding B. (2023). LENet: Lightweight and Efficient LiDAR Semantic Segmentation Using Multi-Scale Convolution Attention. arXiv.

[6] Simonyan K., Zisserman A. (2014). Very Deep Convolutional Networks for Large-Scale Image Recognition. arXiv.

[7] Szegedy C., Liu W., Jia Y., Sermanet P., Reed S., Anguelov D., Erhan D., Vanhoucke V., Rabinovich A. Going Deeper with Convolutions. Proceedings of the 2015 IEEE Conference on Computer Vision and Pattern Recognition (CVPR).

[8] He K., Zhang X., Ren S., Sun J. Deep Residual Learning for Image Recognition. Proceedings of the 2016 IEEE Conference on Computer Vision and Pattern Recognition (CVPR).

[9] Huang S., Sun C., Qi L., Ma X., Wang W. (2017). Rice Panicle Blast Identification Method Based on Deep Convolution Neural Network. Trans. Chin. Soc. Agric. Eng..

[10] Xiao X., Yang H., Yi W., Wan Y., Huang Q., Luo J. (2021). Application of Improved AlexNet in Image Recognition of Rice Pests. Sci. Technol. Eng..

[11] Fan C., He B. (2020). Identification of Rice Diseases and Insect Pests Using Transfer Learning. China Agric. Inform..

[12] Wang H., Li G., Ma Z., Li X. Image Recognition of Plant Diseases Based on Backpropagation Networks. Proceedings of the 2012 5th International Congress on Image and Signal Processing.

[13] Padol P.B., Yadav A.A. SVM Classifier Based Grape Leaf Disease Detection. Proceedings of the 2016 Conference on Advances in Signal Processing (CASP).

[14] Amara J., Bouaziz B., Algergawy A. (2017). A Deep Learning-Based Approach for Banana Leaf Diseases Classification. Datenbanksysteme Für Bus. Technol. Und Web (BTW 2017)—Work..

[15] Rahman C.R., Arko P.S., Ali M.E., Iqbal Khan M.A., Apon S.H., Nowrin F., Wasif A. (2020). Identification and Recognition of Rice Diseases and Pests Using Convolutional Neural Networks. Biosyst. Eng..

[16] Prasanna Mohanty S., Hughes D., Salathe M. (2016). Using Deep Learning for Image-Based Plant Disease Detection. arXiv.

[17] Lu Y., Yi S., Zeng N., Liu Y., Zhang Y. (2017). Identification of Rice Diseases Using Deep Convolutional Neural Networks. Neurocomputing.

[18] Chen J., Chen J., Zhang D., Sun Y., Nanehkaran Y.A. (2020). Using Deep Transfer Learning for Image-Based Plant Disease Identification. Comput. Electron. Agric..

[19] Chen J., Zhang D., Suzauddola M., Nanehkaran Y.A., Sun Y. (2021). Identification of Plant Disease Images via a Squeeze-and-Excitation MobileNet Model and Twice Transfer Learning. IET Image Process.

[20] Woo S., Park J., Lee J.-Y., Kweon I.S. (2018). CBAM: Convolutional Block Attention Module. arXiv.

[21] Hu J., Shen L., Albanie S., Sun G., Wu E. (2017). Squeeze-and-Excitation Networks. arXiv.

[22] Wang Q., Wu B., Zhu P., Li P., Zuo W., Hu Q. (2019). ECA-Net: Efficient Channel Attention for Deep Convolutional Neural Networks. arXiv.

[23] Hou Q., Zhou D., Feng J. Coordinate Attention for Efficient Mobile Network Design. Proceedings of the 2021 IEEE/CVF Conference on Computer Vision and Pattern Recognition (CVPR).

[24] Wei Y., Xiao H., Shi H., Jie Z., Feng J., Huang T.S. (2018). Revisiting Dilated Convolution: A Simple Approach for Weakly- and Semi-Supervised Semantic Segmentation. arXiv.

[25] Sandler M., Howard A., Zhu M., Zhmoginov A., Chen L.-C. MobileNetV2: Inverted Residuals and Linear Bottlenecks. Proceedings of the 2018 IEEE/CVF Conference on Computer Vision and Pattern Recognition.

[26] Sharma P., Gupta U.K., Oza M., Sharma S. Reception—A Deep Learning Based Hybrid Residual Network. Proceedings of the 2019 6th Swiss Conference on Data Science (SDS).

[27] Zhang X., Zhou X., Lin M., Sun J. (2017). ShuffleNet: An Extremely Efficient Convolutional Neural Network for Mobile Devices. arXiv.

[28] Wu X., Zhan C., Lai Y.-K., Cheng M.-M., Yang J. IP102: A Large-Scale Benchmark Dataset for Insect Pest Recognition. Proceedings of the 2019 IEEE/CVF Conference on Computer Vision and Pattern Recognition (CVPR).

[29] Yang S., Xiao W., Zhang M., Guo S., Zhao J., Shen F. (2022). Image Data Augmentation for Deep Learning: A Survey. arXiv.

[30] Hao W., Zhili S. (2020). Improved Mosaic: Algorithms for More Complex Images. J. Phys. Conf. Ser..

[31] Liang D., Yang F., Zhang T., Yang P. (2018). Understanding Mixup Training Methods. IEEE Access.

[32] Yun S., Han D., Chun S., Oh S.J., Yoo Y., Choe J. CutMix: Regularization Strategy to Train Strong Classifiers With Localizable Features. Proceedings of the 2019 IEEE/CVF International Conference on Computer Vision (ICCV).

[33] Li L., Zhang S., Wang B. (2021). Apple Leaf Disease Identification with a Small and Imbalanced Dataset Based on Lightweight Convolutional Networks. Sensors.

[34] Bi C., Xu S., Hu N., Zhang S., Zhu Z., Yu H. (2023). Identification Method of Corn Leaf Disease Based on Improved Mobilenetv3 Model. Agronomy.

[35] Liu Z., Mao H., Wu C.-Y., Feichtenhofer C., Darrell T., Xie S. (2022). A ConvNet for the 2020s. arXiv.

[36] Yu W., Zhou P., Yan S., Wang X. (2023). InceptionNeXt: When Inception Meets ConvNeXt. arXiv.

[37] Xu R., Zhang H., Hu W., Zhang S., Wang X. (2022). ParCNetV2: Oversized Kernel with Enhanced Attention. arXiv.

[38] Ma N., Zhang X., Zheng H.-T., Sun J. (2018). ShuffleNet V2: Practical Guidelines for Efficient CNN Architecture Design. arXiv.

[39] Wang X., Wang Y., Zhao J., Niu J. ECA-ConvNeXt: A Rice Leaf Disease Identification Model Based on ConvNeXt. Proceedings of the 2023 IEEE/CVF Conference on Computer Vision and Pattern Recognition Workshops (CVPRW).

[40] Ghosal S., Sarkar K. Rice Leaf Diseases Classification Using CNN With Transfer Learning. Proceedings of the 2020 IEEE Calcutta Conference (CALCON).

[41] Wang Y., Wang H., Peng Z. (2021). Rice Diseases Detection and Classification Using Attention Based Neural Network and Bayesian Optimization. Expert. Syst. Appl..

[42] Mauri A., Khemmar R., Decoux B., Haddad M., Boutteau R. (2022). Lightweight Convolutional Neural Network for Real-Time 3D Object Detection in Road and Railway Environments. J. Real. Time Image Process.

[43] Charles R.Q., Su H., Kaichun M., Guibas L.J. PointNet: Deep Learning on Point Sets for 3D Classification and Segmentation. Proceedings of the 2017 IEEE Conference on Computer Vision and Pattern Recognition (CVPR).

